# Helium Optically Pumped Magnetometers Can Detect Epileptic Abnormalities as Well as SQUIDs as Shown by Intracerebral Recordings

**DOI:** 10.1523/ENEURO.0222-23.2023

**Published:** 2023-12-04

**Authors:** Jean-Michel Badier, Denis Schwartz, Christian-George Bénar, Khoubeib Kanzari, Sébastien Daligault, Rudy Romain, Sergey Mitryukovskiy, William Fourcault, Vincent Josselin, Matthieu Le Prado, Julien Jung, Augustin Palacios-Laloy, Carron Romain, Fabrice Bartolomei, Etienne Labyt, Francesca Bonini

**Affiliations:** 1 Institut de Neurosciences des Systèmes, Institut National de la Santé et de la Recherche Médicale, Aix Marseille Université, Marseille 13005, France; 2 MEG Departement, CERMEP-Imagerie du Vivant, Lyon 69003, France; 3 CEA-LETI, MINATEC, Université Grenoble Alpes, Grenoble 38054, France; 4 MAG4Health, Grenoble 38000, France; 5 Centre de Recherche en Neurosciences de Lyon, Unité Mixte de Recherche S1028, Centre National de la Recherche Scientifique, Hospices Civils de Lyon, Institut National de la Santé et de la Recherche Médicale, Université Lyon 1, Lyon 69002, France; 6 Department of Functional and Stereotactic Neurosurgery, Hôpital de la Timone, Assistance Publique–Hôpitaux de Marseille, Marseille 3005, France; 7 Department of Epileptology and Cerebral Rythmology, Hôpital de la Timone, Assistance Publique–Hôpitaux de Marseille, Marseille 3005, France

**Keywords:** epilepsy, interictal epileptic discharges, magnetoencephalography, optically pumped magnetometers, simultaneous recording, stereotactic-EEG

## Abstract

Magnetoencephalography based on superconducting quantum interference devices (SQUIDs) has been shown to improve the diagnosis and surgical treatment decision for presurgical evaluation of drug-resistant epilepsy. Still, its use remains limited because of several constraints such as cost, fixed helmet size, and the obligation of immobility. A new generation of sensors, optically pumped magnetometers (OPMs), could overcome these limitations. In this study, we validate the ability of helium-based OPM (^4^He-OPM) sensors to record epileptic brain activity thanks to simultaneous recordings with intracerebral EEG [stereotactic EEG (SEEG)]. We recorded simultaneous SQUIDs-SEEG and 4He-OPM-SEEG signals in one patient during two sessions. We show that epileptic activities on intracerebral EEG can be recorded by OPMs with a better signal-to noise ratio than classical SQUIDs. The OPM sensors open new venues for the widespread application of magnetoencephalography in the management of epilepsy and other neurologic diseases and fundamental neuroscience.

## Significance Statement

We performed a simultaneous recording of ^4^He-OPM and intracerebral EEG and validate for the first time OPM results with signals recorded directly within the brain. We demonstrate that epileptic abnormalities seen on intracerebral electrodes are detected by OPMs with a better signal-to noise ratio than classical magnetoencephalography. This represents a significant step toward the validation of OPM-based recordings for epilepsy diagnosis and for clinical and neuroscience research.

## Introduction

Magnetoencephalography (MEG) is a noninvasive electrophysiological recording technology that source reconstruction methods are applied to to localize brain signals with millisecond resolution. The analysis of magnetic brain activity allows for a deeper understanding of the neural substrates of brain pathologies. In the management of drug-resistant epilepsy, MEG contributes to the success of surgical treatment through noninvasive localization of interictal epileptic discharges (IEDs) and can also guide the implantation of depth electrodes when invasive recordings are required (Fischer et al., 2005; Murakami et al., 2016; Alkawadri et al., 2018; Rampp et al., 2019; Corona et al., 2023).

Still, the spread of MEG is strongly limited by the constraints imposed by the use of superconducting quantum interference devices (SQUIDs; Cohen, 1972; Hämäläinen et al., 1993), requiring cooling at a very low temperature (4.2 K). Thus, the sensors are isolated and enclosed in a fixed array inside a rigid dewar, at least 3 cm from the brain. The critical consequences are a substantial reduction in the magnetic signal amplitude, inhomogeneous coverage in terms of brain source-sensor distance, and the need for total immobility during signal acquisition as well as between recording runs.

The emergence of a new generation of MEG sensors, the optically pumped magnetometers (OPMs; Budker and Romalis, 2007), could overcome these limitations. OPMs are quantum sensors that exploit the interaction between an atomic gas and laser light to obtain very precise measurements of tiny magnetic fields. OPMs do not require extreme cooling and can be placed near the scalp in a wearable system thus allowing the subject’s movement (Johnson et al., 2013; Boto et al., 2017; Iivanainen et al., 2017, Boto et al., 2018, 2022; Seymour et al., 2021). The development and improvement of this new technology has taken a quantum leap in recent years, with evolution in miniaturization and sensitivity (for review, see Tierney et al., 2019; Brookes et al., 2022; Labyt et al., 2022). The first commercially available OPMs, based on Alkali atoms (Budker and Romalis, 2007), have a sensitivity of 20 femtotesla/rootHz (fT/rtHz) in triaxis mode compared with 5 fT/rtHz for SQUID sensors. However, their placement close to the scalp allows a threefold to eightfold increase in signal power (Budker and Romalis, 2007; Iivanainen et al., 2017; Johnson et al., 2013), with a significant neuromagnetic signal enhancement compared with SQUIDs, namely for superficial sources (Boto et al., 2016, 2017; Iivanainen et al., 2017).

New OPMs based on helium atoms (^4^He-OPMs) have been developed (Beato et al., 2018), which have a large dynamic range (up to 250 nanotesla, nT) and a large frequency bandwidth (up to 2 kHz) with negligible heat dissipation (10 mW per sensor). Their properties allow recording in a standard magnetic shielding room, facilitating data acquisition.

A few studies have shown that alkali OPMs can record IEDs comparable with those observed with SQUID-MEG (Feys et al., 2022) or previously obtained EEG (Vivekananda et al., 2020). However, to compare OPM to SQUID-MEG, it is necessary to ensure that both modalities are recording equivalent IEDs. By using intracerebral stereotactic EEG (SEEG) as a ground truth, simultaneously recorded with OPMs and SQUIDs (Badier et al., 2017), we aimed to demonstrate that ^4^He-OPMs can perform as well as SQUID-MEG but at an expected lower cost and with greater convenience. These unique simultaneous recordings allowed selecting similar IEDs to compare SQUID-MEG and ^4^He-OPM and thus evaluate the signal-to-noise ratio (SNR) of both techniques.

## Materials and Methods

**Figure 1.**
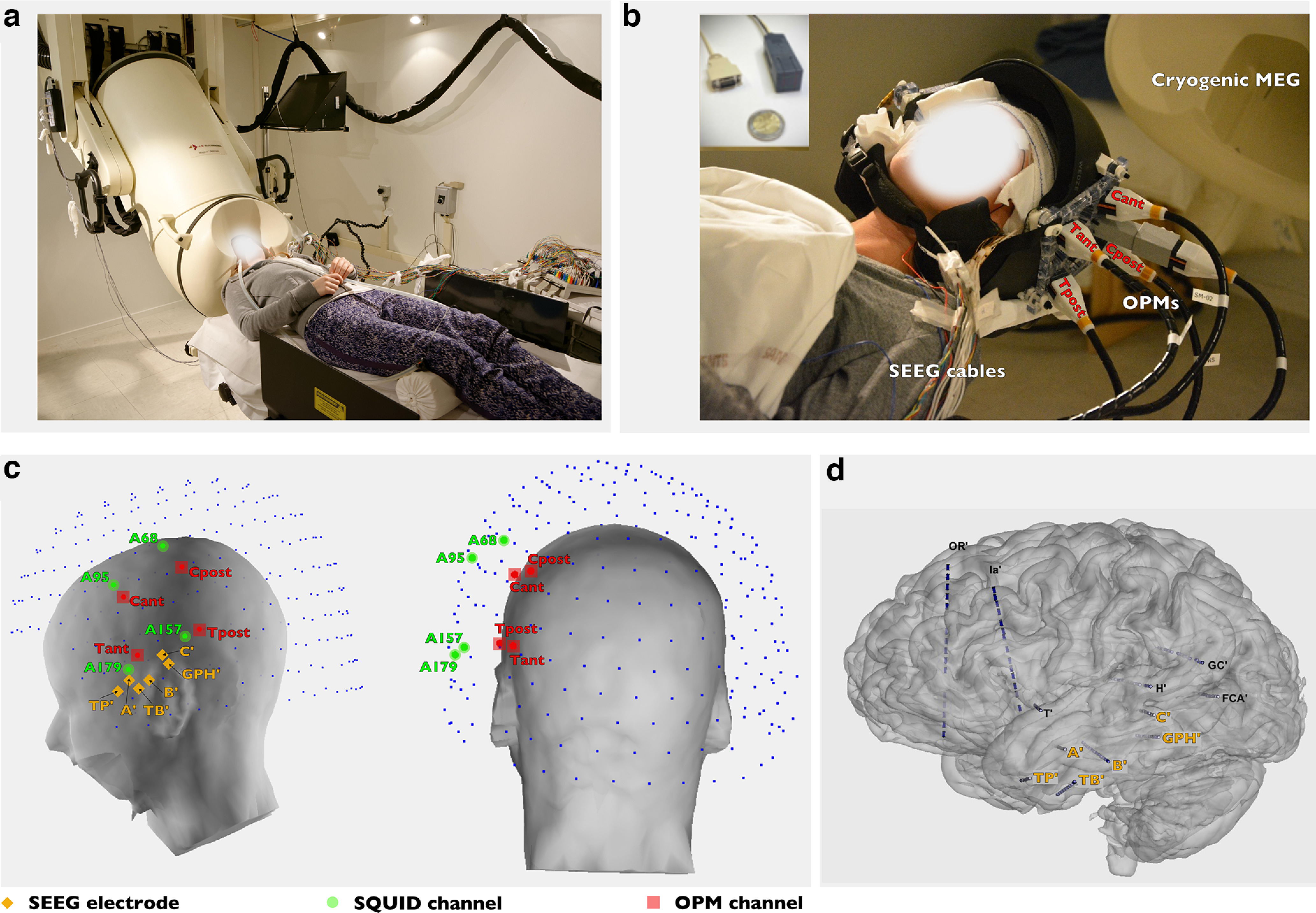
SQUID-MEG, ^4^He-OPM-MEG, and SEEG recording setup. ***a***, Simultaneous SQUID-MEG/SEEG. The classic cryogenic MEG system, measuring 120 × 100 cm and weighing ∼300 kg with 265 SQUID sensors in a fixed array requiring subject immobility during data acquisition. ***b***, Simultaneous ^4^He-OPM-MEG/SEEG: recording configuration composed of four sensors integrated into a wearable helmet placed on the scalp and in contact with the bandage covering the SEEG electrode inserts (the cables and connectors outside the helmet are visible). Insert, A photo of the ^4^He-OPM sensor. ***c***, Three-dimensional reconstruction of the patient's head from MRI, with ^4^He-OPM (red, named Cant, Cpost, Tant, Tpost), SQUID sensors (blue), and SEEG electrodes entry points (orange). The four SQUID sensors (named A95, A68, A179 and A157) closest to the OPMs are in green. Note the distance between the SQUIDs and the scalp (at least 3 cm), whereas the ^4^He-OPMs are in contact with it. ***d***, SEEG implantation consists of 14 intracerebral electrodes, 2 in the right hemisphere (not shown) and 12 in the left hemisphere exploring the whole left temporal structures. A’, Amygdala; TB’, rhinal cortex; C’, posterior hippocampus; GPH’, parahippocampal gyrus; T’, anterior insula/lateral T1; H’, thalamus/Heschl gyrus gyrus; Ia’, anterior insula/F2; FCA’, lingual gyrus; GC’, posterior cingulate/T1; OR’, orbitofrontal cortex/middle frontal sulcus.

The simultaneous recordings were performed inside a two-layer Mu-metal magnetic shielded room (MSR) on a patient with drug-resistant focal epilepsy undergoing SEEG. Fourteen intracerebral electrodes with a total of 119 recording contacts were implanted mainly in the left temporal structures (Fig. 1*d*). These electrodes, and particularly those exploring the anterior medial and lateral temporal regions, disclosed abundant interictal epileptic abnormalities. A first SQUID-MEG/SEEG recording session (Fig. 1*a*) of 20 min at rest was followed by a comparable ^4^He-OPM/SEEG session. The four ^4^He-OPM sensors (2 × 2 × 5 cm) fixed on a rigid helmet (Fig. 1*b*) were placed over the left central and temporal regions, in contact with the bandage covering the scalp (Fig. 1*c*, red dots). The four SQUID sensors closest to the four ^4^He-OPMs sensors were selected for analysis and for comparison with the magnetic activity recorded by the OPM sensors (Fig. 1*c*, green dots). These four SQUIDs were between 2.72 and 3.23 cm away from the OPM sensors.

10.1523/ENEURO.0222-23.2023.f1-1 Figure 1-1 Averaged spike III results. ***A***, ***B***, The averaged signal (6 events) collected during the SQUID-MEG/SEEG simultaneous session. Bipolar averaged SEEG data (***A***). The averaged spikes involve only deep leads of the B’ electrode (left anterior hippocampus). Simultaneous SQUID-MEG averaged data on the four sensors closest to the ^4^He-OPM channels (***B***); no spike was clearly identified. ***C***, ***D***, The averaged signal (6 events) collected during the ^4^He-OPM-MEG/SEEG simultaneous session. Bipolar averaged SEEG data (***C***). Note the similarity between the two intracerebral spikes disclosing the same anatomical location and time course. Simultaneous ^4^He-OPM-MEG averaged data collected on four channels (D). t, Tangential magnetic field (dotted lines); r, radial magnetic field (continuous lines), No spike is clearly identified. The vertical scale is identical to SQUID-MEG data in ***A***, ***B***. Neither SQUID-MEG nor ^4^He-OPM-MEG detected signals linked to the events occurring on B’. Download Figure 1-1, TIF file.

### Patient

The patient was a 33-year-old woman with intractable temporal epilepsy associated with a cavernoma of the left parahippocampal gyrus. An SEEG was performed to define the epileptogenic zone to be removed to study its relationship with the functional areas of language and to evaluate the functionality of the hippocampus and the possible functional risk in terms of memory in case of epilepsy surgery. Most electrodes (12/14) were aimed at an extensive exploration of the left temporal structures, as well as the anterior insula and the orbitofrontal cortex; two electrodes were implanted in the right anterior temporal region. At the end of the SEEG, the epileptogenic zone network could be defined as involving the left mesial temporal structures including the amygdala, hippocampus, rhinal cortex, left parahippocampal cortex and collateral sulcus (the latter being posterior to the cavernoma), and the left temporal pole. The simultaneous recording session was conducted at the end of the long-term video SEEG recording, at J11 from electrode implantation once all clinical data were acquired. Ethical approval was obtained at the Comité de Protection des Personnes Sud Méditerranée I under ID RCB 2020-A01830-39, and the patient gave informed consent. 

### SEEG recordings

SEEG exploration was performed using intracerebral multiple-contacts electrodes placed intracranially with robotic assistance (ROSA, Zimmer Biomet) and intraoperative Mobius Airo CT-scan (Stryker) verification. Small insertion screws (catalog #2023-VG-C-10 or #2023-VG-C-10-15, Alcis) were used to minimize the bulk of the electrodes around the skull and to allow for simultaneous recordings. The electrodes had a diameter of 0.8 mm and contained 10–15 contacts. Each contact consisted of 2-mm-long platinum iridium and was separated from each other by 1.5 mm of insulated material (Alcis). To accurately define the anatomic position of each SEEG contact along the electrode trajectory, a CT-scan/MRI data fusion was performed using the in-house software GARDEL (a Graphical User Interface for Automatic Registration and Depth Electrodes Localization). This MATLAB-based tool is able to coregister the MRI to the CT scan and automatically segment and localize depth electrodes contacts by image processing. The signals were formatted in a bipolar configuration keeping only noncontiguous bipolar channels. Recording was made with a Brain Products BrainAmp DC amplifier. SEEG data have been sampled at 2500 Hz.

### SQUID-MEG recordings

The first recording was made with a SQUID MEG system simultaneously with the SEEG (see above) while the patient was in a supine position. MEG signals were acquired on a 4D Neuroimaging 3600 whole-head system at a sampling rate of 2034.51 Hz with a total of 248 magnetometers. Additionally, three magnetometers and nine gradiometers were used for noise compensation. Electrocardiographic and the electrooculographic activity was recorded on bipolar EEG channels.

### ^4^He-OPM recordings 

The second recording was made with a prototype of five ^4^He-OPM sensors simultaneously with SEEG, also while the patient was in a supine position. The ^4^He-OPMs are sensors that measure the brain magnetic field along the three axes with continuous self-compensation of the magnetic field on all axes. The magnetic field measurement relies on a measure of the variation of the light absorption caused by the deviation of the electronic spin of ^4^He atoms from the state originally set by laser pumping. Technical information and physical principles used in our sensors can be found in a previous publication (Fourcault et al., 2021) and are summarized below. The OPM used in this study is based on parametric resonance of helium-4 metastable atoms at near zero magnetic field (DuPont-Roc, 1971; Beato et al., 2018). The cell containing the ^4^He gas is a cylinder 1 cm in diameter and 1 cm in height. This cell, placed at the bottom of the sensor, is surrounded by small three-axis Helmholtz coils that are used to apply both the radiofrequency (RF) fields and the compensation fields (see below). A high-frequency (HF) discharge (excited between 10 and 20 MHz and consuming ∼10 mW power) excites the ^4^He atoms from their ground state to the metastable triplet state, which has three Zeeman sublevels. Selective optical pumping (with a linearly polarized beam tuned on the D0 line at 1083 nm) was performed to prepare a macroscopic magnetic moment on the gas, which evolves in the magnetic field created by the brain. In our OPMs, to derive a vector measurement of the three components of the magnetic field, two RF fields are applied to the gas, B_Ω_cosΩt along one tangential *x*-axis and B_ω_cosωt along the radial *y*-axis. Both are orthogonal to the polarization of the pump laser beam. Using this scheme, first introduced by DuPont-Roc (1971), three resonance signals were detected on the transmitted pump light at *Ω*, *ω*, and *ω ± Ω*. At first order, the amplitude of each resonance is respectively proportional to one of the three components of the magnetic field to be measured (Bx, By, and Bz, respectively). 

Each sensor is operated in a closed-loop mode on the three axes. This consists in continuously cancelling the three components of the magnetic field by applying an opposite field with the three-axis Helmholtz coils. The value of each magnetic field component is deduced from the current injected in the compensation coil. In this way, the sensor becomes self-calibrated, that is, its output can only be affected by variations of the transfer function between the current and magnetic field set by the coil geometry and not by other operating parameters (light intensity, HF power, etc.). This closed-loop mode suppresses the cross-axis effects (Cohen-Tannoudji et al., 1970a,b; DuPont-Roc, 1970, 1971) by which the measurement of one axis becomes dependent on the field along another axis. This phenomenon has been recently referred as the Cross-Axis Projection Error (CAPE; Borna et al., 2022), yielding both phase errors and a tilt of the sensing axis. Previous studies with alkali OPM operated in an open-loop mode have characterized an axis tilt of 3.3°/nT at low frequencies (Borna et al., 2022), and offsets change as small as ±1.5 nT resulted in gain errors of ∼4% (Boto et al., 2018). Another study, focusing on an active shielding system, reported that the calibration errors of the alkaline OPM were three to four times higher when the ambient field was not compensated (Iivanainen et al., 2019). Thereby, ^4^He OPM is the first sensor, to our knowledge, to provide a measurement of the magnetic field components in a closed-loop mode along the three axes, guaranteeing the reliability of the measurement and avoiding any CAPE. Another important advantage of closed-loop operation is the possibility of broadening the dynamic range well above the atomic line width. A dynamic range of ±250 nT is currently achieved for our ^4^He OPMs. The sensitivity of our magnetometer operating in the closed-loop triaxial mode is better than 50 fT/Hz^1/2^ on two of the three axes (the radial and one tangential) with a bandwidth going from DC to 2 kHz. The sensitivity of the third axis was 200 fT/Hz^1/2^. This axis was not used in further data processing steps in the present study.

However, if the closed-loop mode avoids CAPE, it has some unwanted consequences because of the cross talks that unavoidably exist between the sensors within the OPM array. This problem can be solved by appropriate postprocessing as far as the cross talks are appropriately characterized (Fourcault et al., 2021, provides a detailed description). The measured cross talk matrix for an array of four ^4^He OPM sensors with only 2 mm spacing, which corresponds to an extremely unfavorable situation compared with a real OPM MEG setup, revealed low cross talk (<10%) and showed a good agreement with the estimated matrix from the Biot–Savart calculations. Knowing this cross talk matrix, minor cross-talk-related errors are corrected in the measurement by adequate postprocessing. OPM data have been sampled at 11161 Hz and downsampled to 4 kHz.

### OPM, SQUID, and SEEG spatial coregistration

Spatial coregistration was performed for both sessions using a 3D Polhemus digitizer, based on three fiducial markers (nasion and left and right preauricular points). The quality of the coregistration was checked using the digitization of the facial mask. Following the standard procedure for 4D neuroimaging, the position of nasion and both tragi were digitized to allow the construction of the patient frame. The positions of five coils located on the subject’s head were also digitized. Activations of these five coils before and after recording allowed to determine the location of the MEG sensors within the patient frame. For the ^4^He OPM-MEG/SEEG session, the same three fiducial markers were digitized in the same reference frame. In this case, the sensors were linked to the subject’s head by the ^4^He OPM headset. This headset was positioned on the subject’s head before insertion of the ^4^He OPM sensors, and the corresponding slots were digitized giving the location of each ^4^He OPM sensor within the same patient reference frame. Finally, both types of sensors were localized in the same reference frame, that is, the subject’s head. It was then possible to select the SQUID sensors closest to each ^4^He OPM based on Euclidean distance. A preliminary SQUID MEG recording, performed several months before in the context of a noninvasive presurgical evaluation, allowed us to appreciate the typical topography of IEDs of this patient and therefore to choose the placement of OPMs. The four OPM sensors were placed according to this topography, with the constraint of the limited number of locations on the helmet. The signal recorded from OPMs therefore does not necessarily correspond to the maximum amplitude that can be recorded on this patient. The signal obtain from each OPM is compared with the one recorded from the closest SQUID sensor. This one, given the geometry of the array of sensors, lies on a straight line starting from a point near the center of the head and passing through the OPM sensor. This makes it the best choice for comparing the two signals.

### Data processing

**Figure 2.**
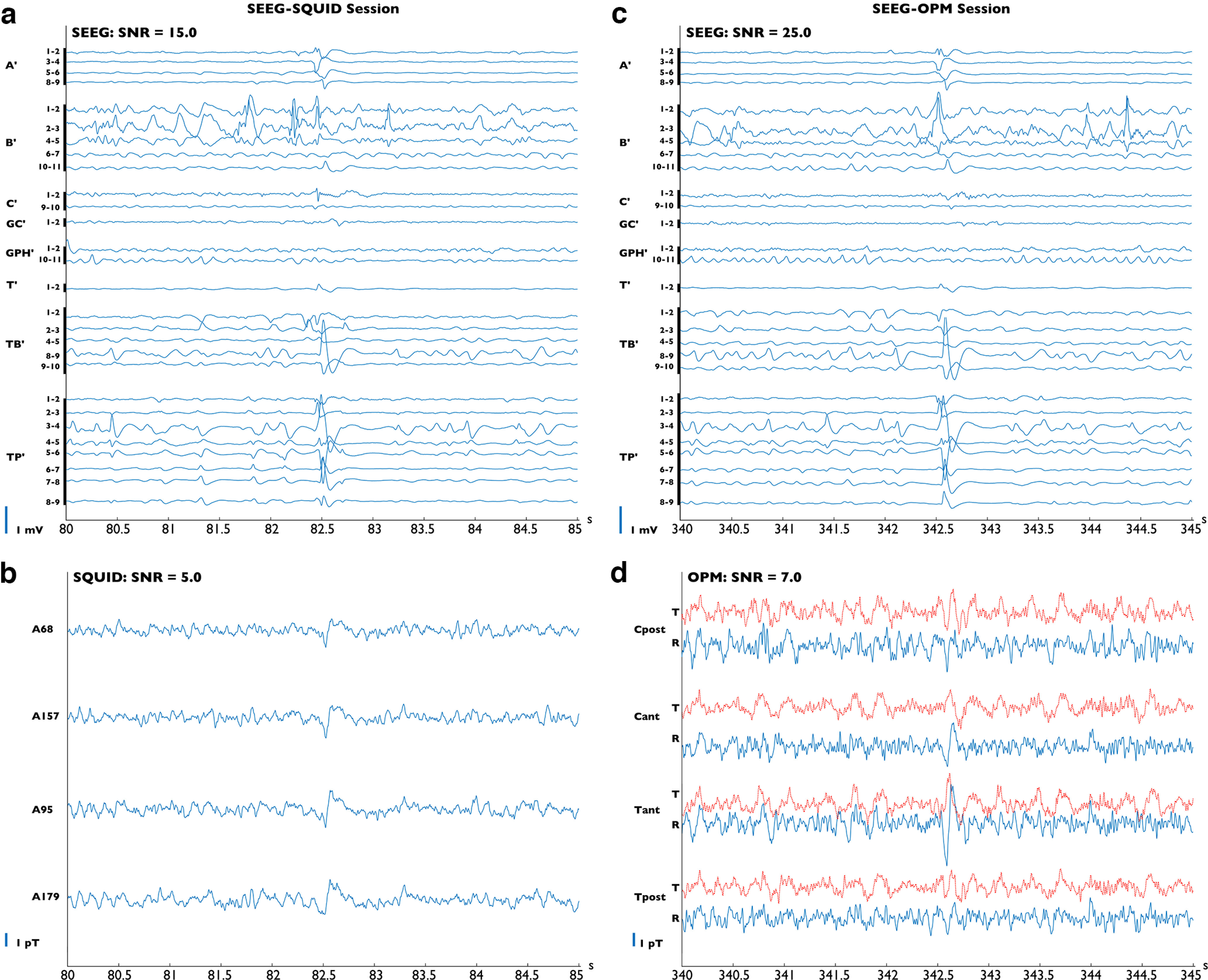
Individual spike results. ***a***, ***b***, The signal collected during the SQUID-MEG/SEEG simultaneous session. Bipolar SEEG data (***a***). The name of the SEEG electrodes and the recording contact label are shown. The labels range from 1 (deeper location) to 11 (more superficial location). The spike (SNR = 14.9) clearly involves both deep and more superficial structures (amygdala, anterior and posterior hippocampus, third anterior temporal gyrus, and temporal pole). Simultaneous SQUID-MEG data collected on the four sensors closest to the ^4^He-OPM channels (***b***); an interictal epileptic spike appears at ∼82.5 s, with a peak-to-peak amplitude of 1.1 pT. ***c***, ***d***, The signal collected during the ^4^He-OPM-MEG/SEEG simultaneous session. Bipolar SEEG data (***c***). Note the similarity between two intracerebral spikes disclosing the same anatomic location and time course (SNR = 24.8). Simultaneous ^4^He-OPM-MEG data collected on four sensors (***d***). t, Tangential magnetic field (red lines); r, radial magnetic field (blue lines). A spike appears at 342.5 s with a peak-to-peak amplitude of 2,5 pT. The vertical scale is identical to that of the SQUID data in ***a***, ***b***.

**Figure 3.**
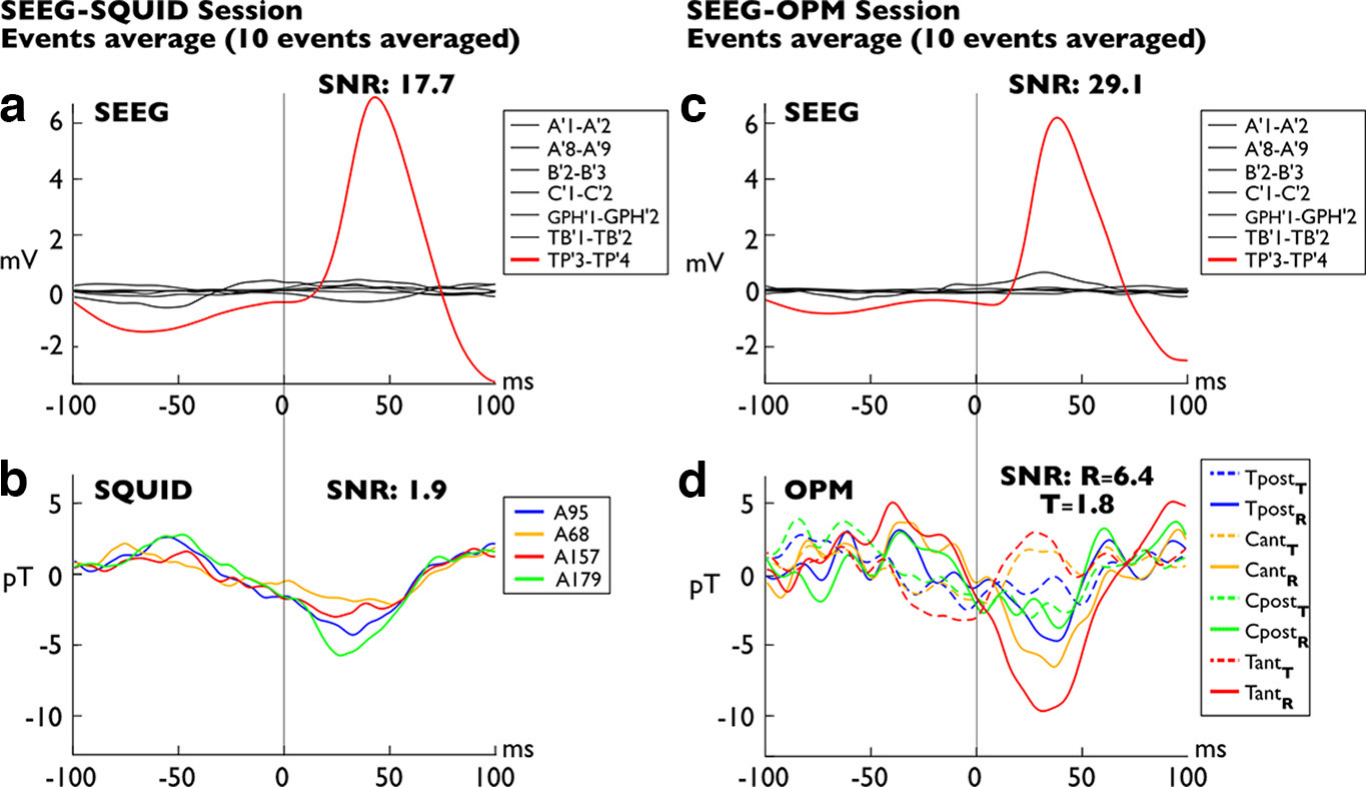
Averaged Type I spikes (*n* = 10). ***a***, ***b***, The averaged signal collected during the SQUID-MEG/SEEG simultaneous session. ***c***, ***d***, The averaged signal collected during the ^4^He-OPM-MEG/SEEG simultaneous session. The names of the SEEG electrodes and recording contacts are shown. Contacts range from 1 (deeper location) to 9 (most surface location). Bipolar averaged SEEG data for the SQUID session (***a***). The averaged spike involves only the bipolar TP’3–TP’4 recording. Simultaneous SQUID-MEG averaged data on the four sensors closest to the ^4^HeOPM sensors (***b***); a small interictal spike appears ∼25 ms with a peak-to-peak amplitude of 8 pT. Bipolar averaged SEEG data for the OPM session (***c***). Note the similarity between the intracerebral spikes of the two sessions, disclosing the same anatomic location and time course. ^4^He-OPM-MEG averaged data collected on four sensors, T_pos_, C_ant_, C_post_, and T_ant_ (***d***). pos, Posterior; ant, anterior. The subscript indicates the orientation. T, Tangential magnetic field (dashed lines); R, radial magnetic field (solid lines). A spike appears at 35 ms with a maximum peak-to-peak amplitude of 15 pT. The vertical scale is identical to that of the SQUID data in ***a***, ***b***. Extended Data Figures 1-1 and 2-1 show more details on spikes involving medial temporal structures.

**Figure 4.**
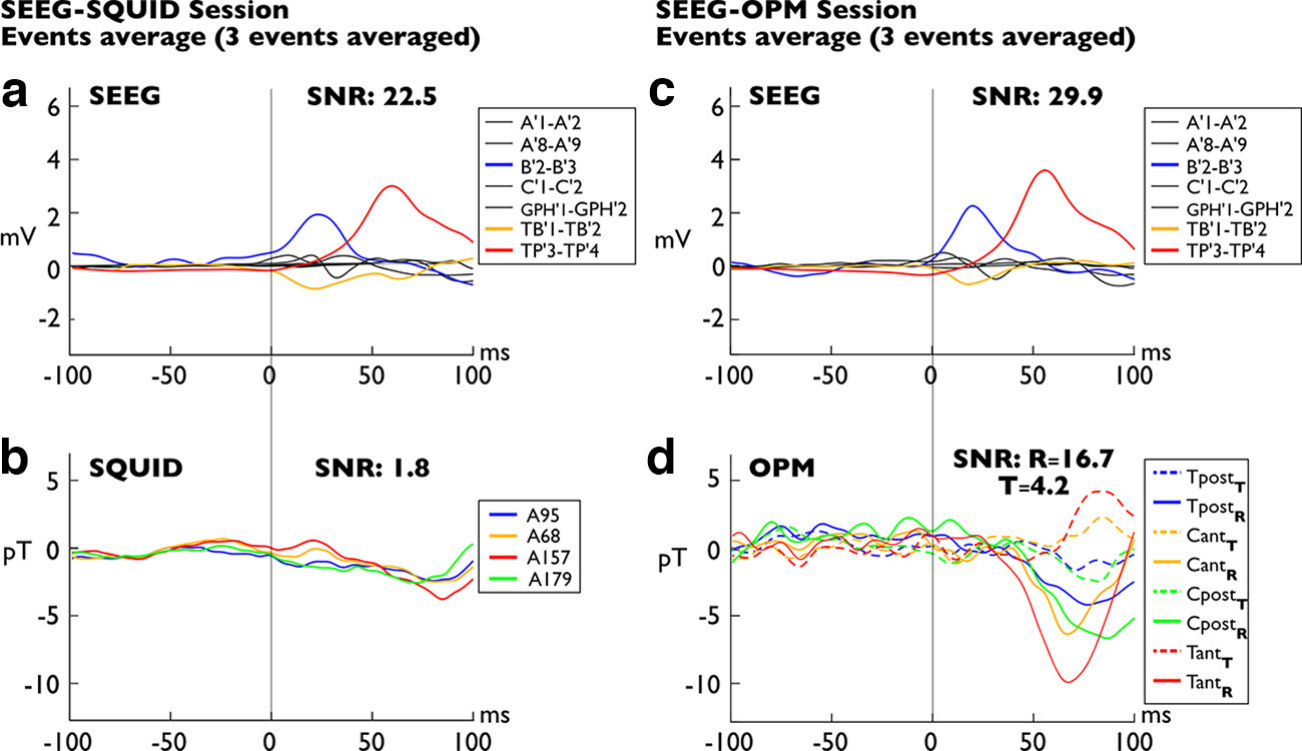
Averaged Type II spikes (*n* = 3). Arrangement of figure is similar to that of Figure 3. ***a***, Bipolar averaged SEEG data for the SQUID session. The spikes involve a larger network encompassing TP’, TB’ and B’ electrodes (medial and lateral temporal pole, anterior hippocampus, third anterior temporal gyrus). TB’, Rhinal cortex. ***b***, Simultaneous SQUID-MEG averaged data on the four sensors closest to the ^4^He-OPM sensors; a very faint spike appears at ∼75 ms with a peak-to-peak amplitude of 4 pT. ***c***, Bipolar averaged SEEG data for the OPM session. Note the similarity between the intracerebral spikes of the two sessions, disclosing the same anatomic location and time course. ***d***, ^4^He-OPM-MEG averaged data collected on four sensors, T_pos_, C_ant_, C_post_, T_ant_. pos, Posterior; ant, anterior. The subscript indicates the orientation. T, Tangential magnetic field (dashed lines); R, radial magnetic field (solid lines). A spike appears at 75 s with a peak-to-peak amplitude of 9.3 pT maximum. The vertical scale is identical to that of the SQUID data in ***a***, ***b***. Extended Data Figures 1-1 and 2-1 show more details on spikes involving medial temporal structures.

For noise correction, a noise compensation was performed for SQUIDs thanks to recordings from reference sensors (magnetometers and gradiometers). Noise compensation is obtained by subtracting the contribution of the noise measured by the references for each sensor. As there are not enough sensors to perform this, there is no such compensation for the OPM. In that case noise cancellation is only performed by filtering (bandpass filter 2–70 Hz and a notch filter at 50 Hz). For downsampling and temporal registration, because the data were acquired with different devices, postprocessing was necessary to temporally register the simultaneous recordings with the same sampling rate and the same number of samples. To do so, randomly distributed triggers were sent to both kinds of MEG sensors (SQUID or OPM) and SEEG. Data were then processed by an in-house MATLAB function to match the sequence of triggers and to resample the data (from SEEG to an MEG time frame). The resampling procedure did not result in differences in delays larger than one sample. The end results for each session (SQUID-SEEG and OPM-SEEG) were two files that contained the same number of samples with synchronized SEEG and MEG data. For filtering, all data were bandpass filtered at 2–70 Hz; a notch filter at 50 Hz was added. For SNR computation, to investigate the amplitude of events, the SNR was computed as the following ratio: the max amplitude of the event divided by the SD of a baseline (2 s of signal before the event for the single event presented in Fig. 2 and 500 ms for the averages in Fig. 3 and Fig. 4).

10.1523/ENEURO.0222-23.2023.f2-1 Figure 2-1 Averaged spike IV results. ***A***, ***B***, The averaged signal (10 events) collected during the SQUID-MEG/SEEG simultaneous session. Bipolar averaged SEEG data (***A***). The averaged spikes involve only deep leads of the GPH’ (left posterior hippocampus) electrode. Simultaneous SQUID-MEG averaged data on the four sensors closest to the ^4^He-OPM channels (***B***). No spike is clearly identified. ***C***, ***D***, The averaged signal (10 events) collected during the ^4^He-OPM-MEG/SEEG simultaneous session. Bipolar averaged SEEG data (***B***). Note the similarity between the two intracerebral spikes disclosing the same anatomical location and time course. Simultaneous ^4^He-OPM-MEG averaged data collected on four channels (***D***). t, Tangential magnetic field (dotted lines); r, radial magnetic field (continuous lines). No spike is clearly identified. The vertical scale is identical to the SQUID data in ***A***, ***B***. Neither SQUID-MEG nor ^4^He-OPM-MEG detected signals linked to the events occurring on GPH’. Download Figure 2-1, TIF file.

For automatic extraction of events of interest, we had to compare data recorded in the two separate sessions. To do so, we used a procedure to identify identical events across recordings. As a first step, an expert neurologist (F.Bonini) manually selected four kinds of events of interest from the SEEG recording of the SQUID session. The selection was done using a montage of one bipolar SEEG derivation per explored brain area. These reference events were chosen to be representative of IEDs involving lateral temporal structures (spike I) and IEDs involving both medial and lateral temporal structures (spike II). We used the function findsignal in MATLAB (version 202b) to find similar intracerebral events for both the SQUID session and the OPM session. Three to 10 events were kept for averaging. From this set of events, we chose a representative occurrence of spike II to illustrate a single event (Fig. 1).

## Results

As a main result, the analysis of the simultaneous recordings globally shows that the activities recorded by the intracerebral electrodes placed in the left temporal pole are detected on the scalp surface by both systems, conventional SQUID-MEG and OPM-MEG (Figs. 2, 3, 4). Notably ^4^He-OPM-MEG recordings showed an SNR better than that of SQUID-MEG.

Single interictal events (epileptic spikes), are visible on both OPM and SQUID recordings. Figure 2 presents single epileptic spikes with the corresponding SEEG traces. Using bandpass filtering (2–70 Hz) and a notch filter (50 Hz) with no further processing, the ^4^He-OPM-MEG can clearly record, with a high SNR, the IEDs identified with the SEEG electrodes. A single epileptic spike arising from the left temporal pole and the adjacent anterior third temporal gyrus (Fig. 2*c*, intracerebral traces) is distinctly identified on the scalp by the ^4^He-OPM-MEG (SNR = 7, peak-to-peak amplitude = 5 pT) (Fig. 2*d*). In comparison, the four closest SQUID-MEG sensors, using advanced denoising based on reference sensors, detect an equivalent intracerebral spike (Fig. 2*a*, visible on simultaneous SEEG) but with a lower SNR of five (Fig. 2*b*). This can be explained by the reduced distance from the neuronal sources of ^4^He-OPM-MEG, yielding increased signal power compared with SQUID-MEG (Labyt et al., 2019), even if this should be weighed against the fact that the SEEG has a slightly higher SNR of the same order of magnitude. Furthermore, it is interesting to note that different time courses and polarities are clearly identifiable, depending on the location of the channel and the orientation of the magnetic field (Fig. 2*d*). The ^4^He-OPM sensors are natively sensitive to two orthogonal orientations of the magnetic field so that two different signals corresponding to the tangential and radial components (Fig. 2*d*, red lines and blue lines, respectively) are output for each sensor.

These results observed on single spikes are even more evident when comparing the average of ^4^He-OPM-MEG and SQUID-MEG signals (Fig. 3). To compare a sufficient number of equivalent epileptic events recorded by OPMs and SQUID, we manually identified, using a selective SEEG electrodes montage, four different types of epileptic spikes recorded in the two separate simultaneous sessions (see above, Materials and Methods; Extended Data Figs. 1-1, 2-1). These IEDs have been subsequently automatically extracted from the entire time series and averaged by type. Two types of IEDs are illustrated, an average spike arising from the left temporal pole only (Fig. 3*a*,*c*, red line) and an average spike arising from the temporal pole, the third anterior temporal gyrus (minimally), and the anterior hippocampus (Fig. 4*a*,*c*, red, orange, and blue lines, respectively). In both cases, the simultaneous SQUID-MEG averaged data reveal a small deflection (Figs. 3*b*, 4*b*, respectively, −6 pT and 3.7 pT maximum value) in correspondence with the averaged intracerebral spikes (Figs. 3*a*, 4*a*). In contrast, the ^4^He-OPM-MEG averaged data disclose clear averaged spikes (Figs. 3*d*, 4*d*) occurring along with the two averaged intracerebral spikes (Figs. 3*c*, 4*c*). Both ^4^He-OPM-MEG spikes have a higher amplitude (spike I = −9.5 pT; spike II = −10 pT) and a higher SNR (spike I, SNR, *r* = 6.4; spike II, SNR, *r* = 16.7) than those detected in the SQUID-MEG data. Regarding averaged spike II, it is interesting to note that the time course of the OPM signal appears to be correlated with the decay of the temporopolar spike, as recorded by temporal pole (TP’) electrodes 3–4, whereas the hippocampal activity recorded by anterior hippocampus (B’) electrodes 2–3 is not detected by MEG sensors, SQUIDs nor OPMs. On the other hand, variable time courses, polarities, and amplitudes between radial and tangential measurement axes can be observed on the OPM signals, particularly on spike II (Fig. 4*d*). The radial components of the two anterior sensors show a negative deflection, whereas for the corresponding tangential components, the deflection is positive and slightly delayed. This suggests that ^4^He-OPM-MEG provides more information than SQUID-MEG about the spatiotemporal organization of IEDs across the cerebral cortex, thanks to their tangential component.

## Discussion

In this study we report the results of two sets of simultaneous intracerebral recordings of IEDs, one with ^4^He-OPM-MEG and the other one with SQUID-MEG. Using SEEG as reference and ground truth, we correlated the IEDs recorded by intracerebral electrodes with both ^4^He-OPM and SQUID sensors. We obtained the first direct validation of the ability of ^4^He-OPM sensors to record epileptic activities, and we demonstrate that the new ^4^He-OPM system performs better in recording IEDs than the SQUID-MEG system, as evidenced by its higher SNR. Notably, these recordings have been achieved in a regular clinical environment without advanced noise correction. Because of their native 3D measurement of the magnetic field, OPM signals disclose variations in time courses, polarities, and amplitudes between the radial and tangential components of the recorded activity.

Simultaneous acquisition of MEG and intracerebral EEG is a technical feat (Dalal et al., 2009; Santiuste et al., 2008; Dubarry et al., 2014) but can now be performed without major difficulties (Badier et al., 2017). It has several key advantages in comparison with separate acquisition. It allows capturing the same activity at the surface and in depth, avoiding potential differences in brain state and medication. This is particularly important for IEDs that are spontaneous events that can widely vary in extent from one event to the other (Badier and Chauvel, 1995). Simultaneity allows correlating signals across events (Dubarry et al., 2014), and in the current study, allowed finding similar events to be compared in SQUID and OPM sessions based on SEEG topography. With this reliable comparison, we can establish that ^4^He-OPMs are at least as capable of recording epileptic activity as SQUIDs.

Currently, alkali-based OPM are mainly used for MEG recordings in healthy volunteers (for review, see Brookes et al., 2022), and few studies in epileptic patients have been performed (Tierney et al., 2021; Vivekananda et al., 2020; Feys et al., 2022), with one seizure recording recently reported (Feys et al., 2023). Present results strengthen the perspective that OPMs are an accurate and valid alternative to SQUIDs.

Nonetheless, although very promising, clinical adoption of OPMs remains challenging because of some limitations of the current technology. Alkali OPMs have a limited bandwidth (1–100 Hz) and a small dynamic range (5 nT), requiring higher attenuation than a conventional shielded room for SQUID-MEG, demanding a complementary system of magnetic shielding coils to compensate for the remaining magnetic field and to reduce the cross talk. Another challenge posed by the heat dissipation by sensors must be solved, possibly with a helmet design including insulation or an air-flowing system. However, the greater the number of sensors used, the greater the amount of heat dissipated and the risk of the system becoming uncomfortable, probably requiring a more complex active cooling system. Despite these constraints and because of technological advances to overcome them, alkali-based OPMs have given numerous proofs of their good sensitivity to biomagnetic measurement (Labyt et al., 2019; Tierney et al., 2019; Brookes et al., 2022). As evidence of the rapid evolution of the technology, 90-channel OPM systems offering triaxial magnetic field detection have been shown to improve cortical coverage successfully (Boto et al., 2022; Rea et al., 2022). The additional information offered by a vectorial measurement of the magnetic field, also reported in the present study, will be of interest to better characterize the spatiotemporal dynamics of epileptic activity and to interpret clinical data (Iivanainen et al., 2017; Zahran et al., 2022). In our study, the delay of the tangential component (see averaged spike II in Figure 4*d*) is potentially informative on the propagation of the interictal activity, as shown by comparing radial SQUID-MEG and EEG (Merlet et al., 1997).

Helium-based OPMs could overcome some of the limitations of alkali OPM as they can be placed nearest the scalp without any discomfort for the patient because they do not require thermal insulation. ^4^He-OPM are currently the only sensors to be self-compensated on their three measurement axes in a closed-loop operating mode. This ensures the highly reliable measurement of the cerebral magnetic field, with no cross-axis projection errors (Fourcault et al., 2021), and improves the stability of the scale factor of the sensors over time. This also allows for an increased dynamic range (up to 250 nT), larger than the one corresponding to the helium resonance line width, which eliminates the constraint of a strict field nulling system. In this study, ^4^He-OPMs have been used in a standard two-layer MSR in the hospital environment, known to be particularly magnetically noisy. On the other hand, in their current release, ^4^He-OPMs have a worse sensitivity (40 fT/rtHz; Fourcault et al., 2021) compared with alkali OPMs (20 fT/rtHz in tri axis mode; Boto et al., 2022). However, in a recent study in a large group of healthy subjects, the ^4^He-OPMs showed very similar results to the classical SQUID-MEG system because of their shorter distance to the brain (Gutteling et al., 2023). In our study, we observed that ^4^He-OPM can record IEDs with a better SNR compared with SQUIDs. This result, together with the recent data from 18 volunteers, shows that ^4^He-OPMs are able to deliver high-quality brain recordings (Gutteling et al., 2023).

Some limitation of this work comes from the limited number of OPM sensors. Nevertheless, the full coverage of the SQUID system allowed selecting of the colocalized SQUID sensors and ensuring a reasonable comparison. We found that the amplitude of the OPM signals was higher than that of SQUID signals, as expected from both simulations and real data (Boto et al., 2016, 2017; Iivanainen et al., 2017). Still, the MEG IEDs, measured with ^4^He-OPMs and SQUIDs, arose from neocortical structures. We could not show activity from deep structures, such as the hippocampus, on either type of sensor. Sensitivity to deep sources remains a challenge. It will be necessary to use a larger number of sensors to verify whether source separation techniques such as independent component analysis will allow to extract activity from deep sources as previously demonstrated (Pizzo et al., 2019).

With all its advantages, OPM technology could extend the use of MEG to many clinical and research applications. Currently, only very few clinical centers have an MEG facility. By offering more affordability, higher signal sensitivity and bandwidth, and portability without additional equipment, the OPM technology paves the way for the democratization of this unique noninvasive method of high-resolution brain exploration, with a potentially powerful impact on both clinical practice and neuroscience.

## Synthesis

Reviewing Editor: Michael Michaelides, NIDA-NIH Decisions are customarily a result of the Reviewing Editor and the peer reviewers coming together and discussing their recommendations until a consensus is reached. When revisions are invited, a fact-based synthesis statement explaining their decision and outlining what is needed to prepare a revision will be listed below. The following reviewer(s) agreed to reveal their identity: Stefan Rampp, Gareth Barnes.

Reviewer 1
