# Supplementary figures and images for: Helium Optically Pumped Magnetometers Can Detect Epileptic Abnormalities as Well as SQUIDs as Shown by Intracerebral Recordings
Source: eNeuro. 2023 Dec 4;10(12):ENEURO.0222-23.2023. doi: 10.1523/ENEURO.0222-23.2023 (PMC10748329; doi:10.1523/ENEURO.0222-23.2023)

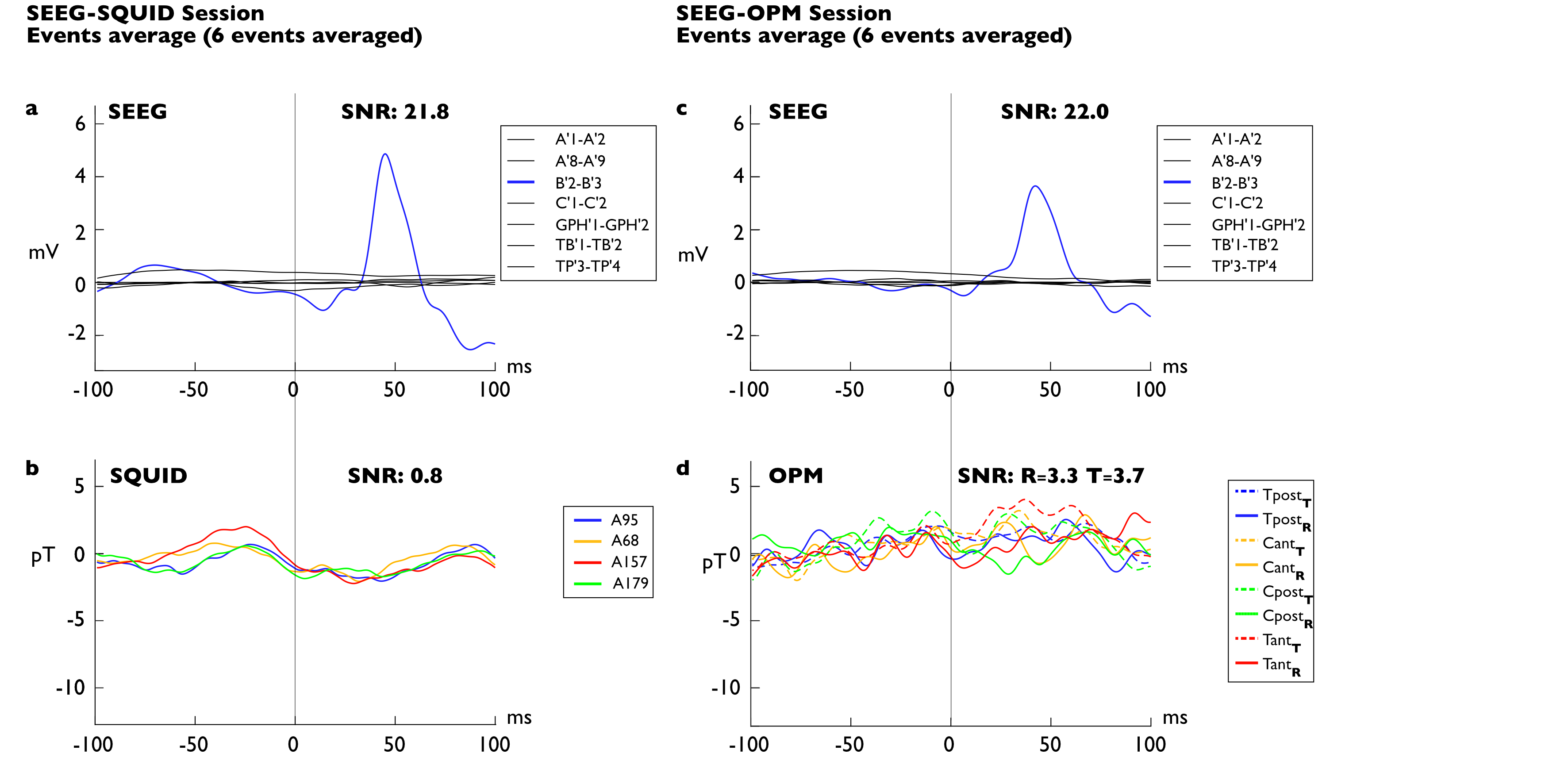

Supplement: Figure 1-1 — Averaged spike III results. A, B, The averaged signal (6 events) collected during the SQUID-MEG/SEEG simultaneous session. Bipolar averaged SEEG data (A). The averaged spikes involve only deep leads of the B’ electrode (left anterior hippocampus). Simultaneous SQUID-MEG averaged data on the four sensors closest to the 4He-OPM channels (B); no spike was clearly identified. C, D, The averaged signal (6 events) collected during the 4He-OPM-MEG/SEEG simultaneous session. Bipolar averaged SEEG data (C). Note the similarity between the two intracerebral spikes disclosing the same anatomical location and time course. Simultaneous 4He-OPM-MEG averaged data collected on four channels (D). t, Tangential magnetic field (dotted lines); r, radial magnetic field (continuous lines), No spike is clearly identified. The vertical scale is identical to SQUID-MEG data in A, B. Neither SQUID-MEG nor 4He-OPM-MEG detected signals linked to the events occurring on B’. Download Figure 1-1, TIF file. [file enu-eN-MNT-0222-23-s01.tif]

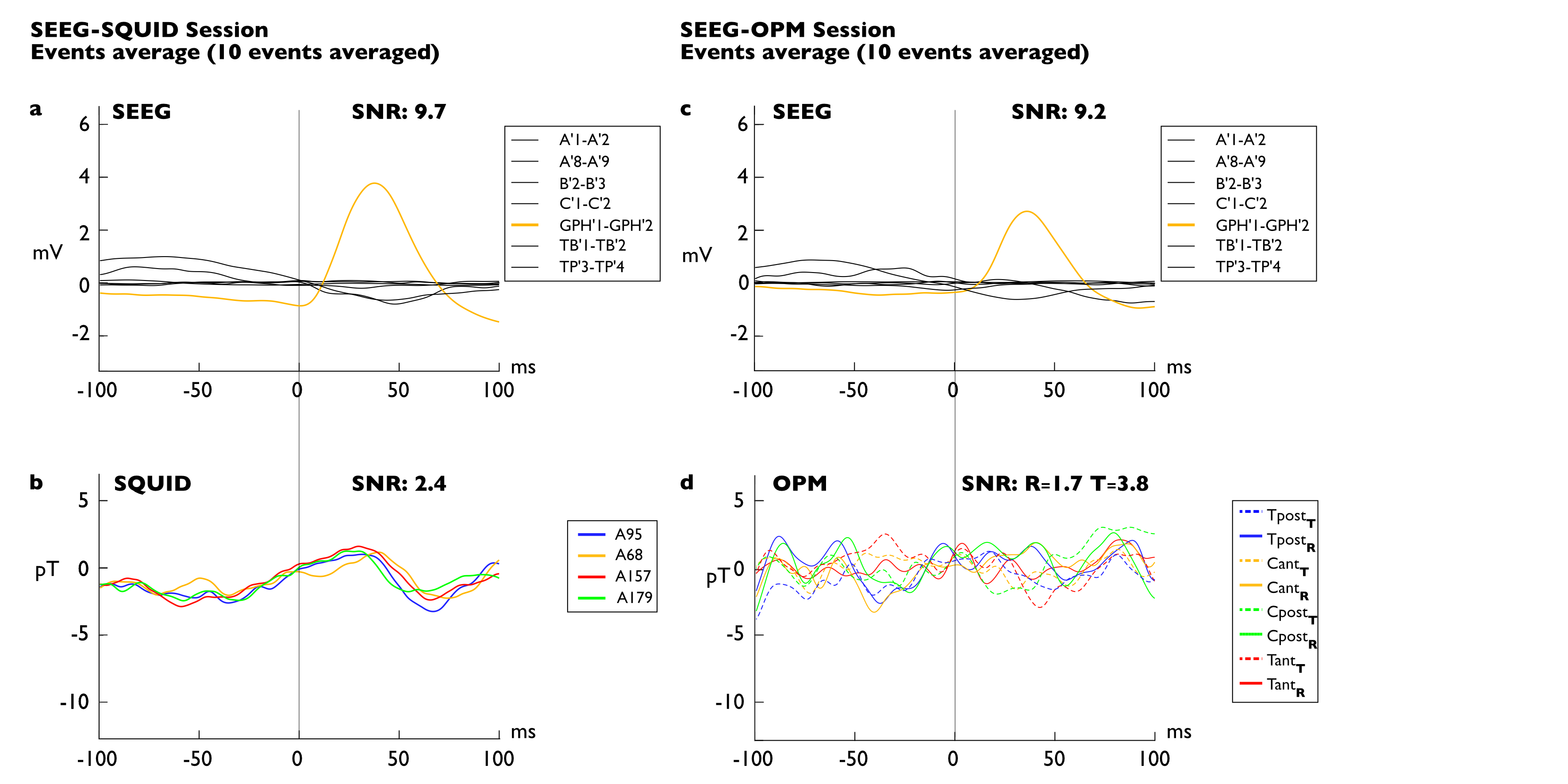

Supplement: Figure 2-1 — Averaged spike IV results. A, B, The averaged signal (10 events) collected during the SQUID-MEG/SEEG simultaneous session. Bipolar averaged SEEG data (A). The averaged spikes involve only deep leads of the GPH’ (left posterior hippocampus) electrode. Simultaneous SQUID-MEG averaged data on the four sensors closest to the 4He-OPM channels (B). No spike is clearly identified. C, D, The averaged signal (10 events) collected during the 4He-OPM-MEG/SEEG simultaneous session. Bipolar averaged SEEG data (B). Note the similarity between the two intracerebral spikes disclosing the same anatomical location and time course. Simultaneous 4He-OPM-MEG averaged data collected on four channels (D). t, Tangential magnetic field (dotted lines); r, radial magnetic field (continuous lines). No spike is clearly identified. The vertical scale is identical to the SQUID data in A, B. Neither SQUID-MEG nor 4He-OPM-MEG detected signals linked to the events occurring on GPH’. Download Figure 2-1, TIF file. [file enu-eN-MNT-0222-23-s02.tif]
